# The *Craterostigma plantagineum* protein kinase CpWAK1 interacts with pectin and integrates different environmental signals in the cell wall

**DOI:** 10.1007/s00425-021-03609-0

**Published:** 2021-04-05

**Authors:** Peilei Chen, Valentino Giarola, Dorothea Bartels

**Affiliations:** 1 Faculty of Natural Sciences, Institute of Molecular Physiology and Biotechnology of Plants (IMBIO), University of Bonn, Kirschallee 1, 53115 Bonn, Germany; 2 College of Life Sciences, Henan Normal University, Xinxiang, 453007 China; 3 Present Address: Department of Genomics and Biology of Fruit Crops, Research and Innovation Centre, Fondazione Edmund Mach, San Michele all’Adige, Italy

**Keywords:** Abiotic stress, Biotic stimuli, Cell wall proteins, Desiccation tolerance, Resurrection plants

## Abstract

**Main conclusion:**

The cell wall protein CpWAK1 interacts with pectin, participates in decoding cell wall signals, and induces different downstream responses.

**Abstract:**

Cell wall-associated protein kinases (WAKs) are transmembrane receptor kinases. In the desiccation-tolerant resurrection plant *Craterostigma plantagineum*, CpWAK1 has been shown to be involved in stress responses and cell expansion by forming a complex with the *C. plantagineum* glycine-rich protein1 (CpGRP1). This prompted us to extend the studies of *WAK* genes in *C. plantagineum*. The phylogenetic analyses of *WAKs* from *C. plantagineum* and from other species suggest that these genes have been duplicated after species divergence. Expression profiles indicate that CpWAKs are involved in various biological processes, including dehydration-induced responses and SA- and JA-related reactions to pathogens and wounding. CpWAK1 shows a high affinity for “egg-box” pectin structures. ELISA assays revealed that the binding of CpWAKs to pectins is modulated by CpGRP1 and it depends on the apoplastic pH. The formation of CpWAK multimers is the prerequisite for the CpWAK–pectin binding. Different pectin extracts lead to opposite trends of CpWAK–pectin binding in the presence of Ca^2+^ at pH 8. These observations demonstrate that CpWAKs can potentially discriminate and integrate cell wall signals generated by diverse stimuli, in concert with other elements, such as CpGRP1, pH_apo_, Ca^2+^_[apo]_, and via the formation of CpWAK multimers.

**Supplementary Information:**

The online version contains supplementary material available at 10.1007/s00425-021-03609-0.

## Introduction

The plant cell wall is a highly organized macromolecular gel-like structure mainly constituted of water, polysaccharides and proteins (Vorwerk et al. 2004). Proteins in cell walls have not only structural and physiological functions but are also essential in signal transduction mediating the transmission of external stimuli to internal response systems (Cosgrove 1997; Caffall and Mohnen 2009; Chen et al. 2020).

Cell wall-associated protein kinases (WAKs), as a class of cell wall proteins, are a subset of the WAK-like (WAKL) superfamily. In *Arabidopsis thaliana*, five *WAK* and twenty-two *WAK-like* (*WAKL)* genes have been identified (He et al. 1999; Verica and He 2002). Analysis of the *A. thaliana* genome sequence revealed that the expansion of the *AtWAK/WAKL* gene family occurred via tandem duplications, segmental duplications and retrotransposon activity (Verica and He 2002). *WAK/WAKL* genes have been described in other plant species (Zhang et al. 2005; Liu et al. 2006; Kaur et al. 2013; Rosli et al. 2013; Hurni et al. 2015; Giarola et al. 2016; Zuo et al. 2019). In rice and apple, the number of *WAKL* genes was expanded up to 125 (Zhang et al. 2005) and 44 (Zuo et al. 2019), respectively. The expansion of the rice *WAKL* genes probably resulted from localized gene duplications (Shiu et al. 2004; Zhang et al. 2005).

The structure of WAK proteins is characterized by a conserved cytoplasmic Ser/Thr kinase domain and by a variable extracellular domain containing EGF (epidermal growth factor) repeats (Kohorn and Kohorn 2012; Kohorn 2015). Calcium is predicted to mediate the dimerization of WAKs involving asparagine residues in the EGF domains (Anderson et al. 2001; Verica et al. 2003). Conserved cysteine residues are involved in the formation of disulfide-bridged complexes (Anderson et al. 2001; Verica and He 2002). Amino acid identities between the extracellular domain of WAKs and extracellular matrix proteins like collagens, tenascins and neurexins suggest that WAK proteins may function in a carbohydrate-rich environment interacting with other proteins or forming oligomers (He et al. 1999). It was demonstrated that the extracellular domains of WAKs bind to pectins via both covalent and ionic bonds (Wagner and Kohorn 2001; Decreux and Messiaen 2005; Decreux et al. 2006June et al. 2019). The extracellular domain of the *A. thaliana* WAK1 has a high affinity for oligo-galacturonides (OGs) in the “egg-box” conformation, which is formed with Ca^2+^ as bridge linking acidic polysaccharides (Grant et al. 1973; Decreux and Messiaen 2005; Cabrera et al. 2008). The galacturonic acid appears to be the key element for WAK–pectin interaction, regardless of the chemical modification of the reducing ends of the OGs (Decreux and Messiaen 2005; Cabrera et al. 2008; Kohorn et al. 2009; Kohorn and Kohorn 2012). AtWAK2 is required for OG-mediated responses of some pectin-regulated genes (Kohorn et al. 2009). In protoplasts, the pectin-activated transcription of vacuolar invertase was triggered by WAK2 and the pectin-induced regulation of mitogen-activated protein kinases was also affected in the *wak2* mutant (Kohorn et al. 2009). The proof that WAKs are the receptors for OGs comes from in vivo domain swap experiments (Brutus et al. 2010; Kohorn and Kohorn 2012). These results demonstrate the interaction between WAKs and pectins/OGs and that OGs are key elements in mediating the activation of downstream signaling pathways upon WAK–OGs interaction. Besides pectins, cell wall glycine-rich proteins (GRPs) were identified as an interaction partner of WAKs (Park et al. 2001; Kohorn and Kohorn 2012; Giarola et al. 2016). Gramegna et al. (2016) discovered that OGs, flg22 and wound treatments prolonged the expression of defense genes, increased H_2_O_2_ accumulation, and enhanced callose deposition in both the over-expressing AtWAK1 and *grp-3* loss-of function mutants. The *grp-3* mutants showed wild-type responses to OGs/flg22/wound treatments when complemented with GRP3 over-expressing plants (Gramegna et al. 2016). Taken together, these observations indicate a positive function (activation) of AtWAK1 and a negative function (repression) of AtGRP3 in the OG/flg22/wound-triggered defense responses (Gramegna et al. 2016). It is not known how OGs and AtGRP3 interact with WAK to initiate the defense responses.

*WAKs/WAKL*s expression suggests that these genes are implicated in different aspects of the plant life cycle. In *A. thaliana*, *AtWAKs* are mainly expressed in the vegetative organs, except for AtWAK4 which is primarily detected in siliques (He et al. 1998; 1999). Of the 22 *AtWAKL*s, *AtWAKL1*, *AtWAKL3* and *AtWAKL5* are expressed mainly in roots and flowers but not in vegetative organs (Verica et al. 2003). *WAKs* from other species also show tissue-specific and developmentally regulated expression patterns (Zhang et al. 2005; Kaur et al. 2013; Zuo et al. 2019). In wheat, the *TaWAKL1* and *TaWAKL2* are mainly expressed in the juvenile stage while *TaWAK1* and *TaWAK3* show stronger expression in adult stages (Liu et al. 2006). The expression of *WAK*s and *WAKL*s is also affected by a range of environmental stimuli. *AtWAK1* is induced by pathogens, exogenous salicylic acid (SA) or its analog 2, 6-dichloroisonicotinic acid (INA) in a NPR (Nonexpressor of pathogenesis-related genes)-dependent manner (He et al. 1998). The SA-inducible *AtWAKs/AtWAKLs* are additionally responsive to wounding (Wagner and Kohorn 2001; Verica et al. 2003). Increasing numbers of *WAKs* have been identified as SA-induced or pathogen-related genes in various species (Liu et al. 2006; Li et al. 2009; Meier et al. 2010; Hu et al. 2014; Hurni et al. 2015; Shi et al. 2016; Saintenac et al. 2018; Zuo et al. 2019; Czajkowska et al. 2019; Gadaleta et al. 2019). Apart from the biotic stress, WAKs also respond to abiotic stressors, such as cold (de Oliveira et al. 2014), heat (Wang et al. 2019), heavy metal (Hu et al. 2014) or dehydration (Giarola et al. 2016).

*Craterostigma plantagineum* is an African resurrection plant, which withstands complete desiccation and revives once water is available (Gaff 1971; Bartels 2005). WAK proteins appear to be involved in the acquisition of desiccation tolerance in *C. plantagineum*. Giarola et al. (2016) observed that *CpWAK1* and *CpWAK2* transcripts decrease during dehydration and accumulate during rehydration. In line with AtWAK1, the CpWAK1 protein binds to pectins and interacts with GRP (Giarola et al. 2016; Jung et al. 2019). Recently, Jung et al. (2019) discovered that the CpGRP1 also binds to pectins with a stronger preference for pectins than CpWAK1. In *C. plantagineum*, the cell wall folding is indispensable during desiccation and rehydration. The apoplastic CpWAK1–CpGRP1 complex is likely to act as a sensor surveilling the pectin status and distinguishing the different signals in the cell wall and then activating downstream signals and finally triggering cell wall folding (Wagner and Kohorn 2001; Maron 2019).

The diversity of the extracellular domain of WAK proteins is the prerequisite for distinguishing different external signals in the cell wall and therefore different WAK members may regulate diverse steps in the same biological process (Wagner and Kohorn 2001). Kohorn (2015) proposed a model to explain how WAKs switch from regulating cell expansion to stress response, but how WAKs distinguish the signals and activate signaling pathways remain unknown. Here, it was shown that CpWAK interacts with pectin and that this interaction modulated by different factors such as pH, Ca^2+^ or other cell wall proteins. The different CpWAK complexes may be required for distinguishing signal transductions in response to different cell wall signals.

## Materials and methods

### Plant material and treatments

*Craterostigma plantagineum* Hochst. (Scrophulariaceae) was grown according to Bartels et al. (1990). Plant relative water content was calculated according to Bernacchia et al. (1996) and used to monitor the dehydration status during dehydration experiments. Mature *C. plantagineum* plants were subjected to dehydration by withholding water until the RWC of plants was around 2% (desiccated). Desiccated plants were rehydrated for two days (RWC = 80%). Leaves were collected at different time points during the dehydration and rehydration treatments. For other treatments, detached *C. plantagineum* leaves were incubated in water, 1 mM salicylic acid (SA) or 100 µM methyl jasmonic acid (MeJA) for 1, 3, 6, 24 and 48 h. After treatments, samples were frozen in liquid nitrogen and stored at −80 °C for further analyses.

### Extraction of genomic DNA

Plant tissues (50–200 mg) were ground to a fine powder in liquid nitrogen. Then, the powder was homogenized in 300 μl 2 × lysis buffer (0.6 M NaCl; 0.1 M Tris–HCl, pH 8.0; 40 mM EDTA, pH 8.0; 4% (w/v) sarcosyl; 1% (w/v) SDS), 300 μl 2 M urea and 600 μl of phenol/chloroform/isoamyl alcohol (25/24/1). The mixture was centrifuged for 10 min (14,000*g*, RT) and the aqueous supernatant was collected. DNA was precipitated by mixing the supernatant with 0.7 volume of isopropanol and subsequent centrifugation for 15–20 min (14000*g*, 4 °C). After washing the pellet twice with 70% (v/v) ethanol, the air-dried DNA pellet was dissolved in TE buffer (10 mM Tris–HCl; 1 mM EDTA; pH 8.0) containing 20 μg/ml RNase A. RNAs in the DNA samples were removed after 5 min of incubation at 37 °C.

### Transcript analysis

Total plant RNA was extracted according to Valenzuela-Avendaño et al. (2005). First-strand cDNA synthesis was performed as described by Hou and Bartels (2015). The first-strand cDNA was used directly for PCR or stored at −20 °C until use. All primers were synthesized by Eurofins MWG Operon (Ebersberg, Germany) and are listed in Table S1. The PCR was performed as follows: 95 °C 3–5 min for initial denaturation; 95 °C 30 s for cycling denaturation; 50–65 °C 45 s for primer annealing; 72 °C for extension using Taq polymerase (1 min/kb); recycle from step 2 for 21–35 times; 72 °C 5–10 min for final extension; 4 °C for holding the samples until they were collected; The annealing temperature and cycle numbers were determined empirically for each PCR.

### Gene cloning, protein expression and purification

The *CpWAK* genomic sequences were obtained by PCR from genomic DNA isolated from *C. plantagineum* leaves. The primers used are shown in Table S1. The PCR products were cloned into pJET 1.2 vectors using the CloneJET PCR Cloning Kit (Thermo Fisher Scientific, St LeonRot, Germany) and the PCR fragments were sequenced.

The cDNA fragments encoding CpWAK1EX (amino acids 31–315), CpWAK2EX (amino acids 37–333), R-1 (amino acids 31–160), R-2 (amino acids 161–315) and R-3 (amino acids 31–220) (Fig. 3a) were amplified from a CpWAK1 cDNA clone (Giarola et al. 2016) using primers to add an *Xho*I site at the 3′ end (CpWAK1_*Xho*I_R, CpWAK2_*Xho*I_R, R-1, R-2-rev, R-3, Table S1). An *Nco*I site is already present in the sequences of CpWAK1, CpWK2, R-1 and R-3. An *Nco*I site was added at the 5′ end of the R-2 sequence with the primer, R-2-for (Table S1). The *Nco*I/*Xho*I fragments were cloned into the expression vector pET28a( +) (Novagen, Darmstadt, Germany) and transformed into BL21 (DE3) *E. coli* cells (Amersham Pharmacia Biotech, Piscataway, NJ, USA). The fusion constructs, pET28_CpGRP1His and pET28 CpGRP1_N-terminal His, were provided by Giarola et al. (2016) and Jung et al. (2019), respectively. Over-expression and purification of the fusion proteins were performed as described by Jung et al. (2019).

### Cell wall protein extraction and protein analysis

Cell wall protein extraction was carried out as described by Printz et al. (2015) in protocol 3 with minor modifications. After concentration using Amicon^®^ Ultra centrifugal filter, the CaCl_2_ and LiCl fractions of cell wall proteins were precipitated with 4 volumes of cold acetone at −20 °C for at least 30 min or overnight. Cell wall proteins were collected by centrifugation at 15000*g* at 4 °C for 15 min and then the pellet was dried under a hood to eliminate acetone residues. Cell wall protein pellets were dissolved in a minimal volume of 1 × Laemmli buffer (Laemmli 1970). The protein concentration was determined according to Bradford (1976). Equal amounts of cell wall proteins from different samples were separated by 12% (w/v) SDS–polyacrylamide gel electrophoresis (PAGE) (Laemmli 1970). The separated proteins were visualized by Coomassie blue G-250 (Zehr et al. 1989) or silver staining (Mortz et al. 2001). Western blot analysis was performed according to Towbin et al. (1979). The polyclonal antibodies were diluted 1:5000 for the detection of CpWAKs and CpGRP1 (Giarola et al. 2016).

### Pectin extraction and estimation

Pectin was extracted using CDTA (1, 2-cyclohexanediaminetetraacetic acid) according to Cornuault et al. (2014). The galacturonic acid content of the CDTA fraction was determined as described in Verma et al. (2014) with a galacturonic acid standard curve obtained with commercial polygalacturonic acid (Sigma 81325) (Fig. S1).

### Enzyme-linked immune sorbent assay (ELISA)

The ELISA binding assay was performed according to Decreux and Messiaen (2005) with modifications. Nunc Maxisorp flat-bottom plates (Invitrogen, CA, USA) were coated with pectin solution (25 µg well^–1^) at 4 °C overnight. Non-specific binding sites were blocked for 2 h at RT with 100 µL of blocking solution (3% (w/v) low fat dried milk, 20 mM Tris–HCl, 150 mM NaCl). The wells were incubated for 2 h at RT with 50 µL of purified His-tagged recombinant protein in binding buffer (1% (w/v) low fat dried milk, 20 mM Tris–HCl, 150 mM NaCl, with or without 2 mM CaCl_2_) after removing the blocking solution. The wells were washed four times with wash buffer (20 mM Tris–HCl, 150 mM NaCl) and incubated with 50 µL of anti-His-tag antibody (1:10,000) (Invitrogen) or anti-WAK antibody (1:2500) or anti-GRP antibody (1:5000) (Giarola et al. 2016; Jung et al. 2019) in incubation buffer (1% (w/v) low-fat dried milk, 20 mM Tris–HCl, 150 mM NaCl) for 1 h at RT. After washing the wells four times, 50 µL of goat anti-rabbit IgG peroxidase antiserum (1:10,000) (Sigma, A9169) prepared in incubation buffer was added and incubated for 1 h at RT. After washing the plates six times, the bound recombinant protein was visualized in the presence of the substrate TMB (3,3′,5,5′-tetramethylbenzidine) (Sigma, T2885). The absorbance was measured at 450 nm after sufficient colour development in the dark and the reaction was stopped by adding 50 µL of 10% (v/v) phosphoric acid. The pH of all the solutions and buffers used are 4, 5, 6, 7 or 8. In the competitive ELISA binding assay, the recombinant proteins were pre-mixed with each other for 1 h at RT. The mixture was loaded on the pectin-coated and blocked wells and incubated for 2 h at RT. After washing the wells, the plate was incubated with anti-WAK or anti-GRP antiserum. Then, the immobilized recombinant protein was detected after being incubated with goat anti-rabbit IgG peroxidase antibody and visualized with TMB as described above.

### DNA sequence, phylogenetic and gene structure analyses

DNA sequencing was performed by GATC Biotech (https://www.gatc-biotech.com/en/index.html). Protein sequence alignments were done using Clustal Omega (https://www.ebi.ac.uk/Tools/msa/clustalo/). Protein domains were identified using NCBI CD-Search tool (http://www.ncbi.nlm.nih.gov/Structure/cdd/wrpsb.cgi) (Marchler-Bauer et al. 2011), SMART tool (http://smart.embl-heidelberg.de/)(Letunic et al. 2017) and TMHMM Server v. 2.0 (http://www.cbs.dtu.dk/services/TMHMM/).

The WAK homologs (Table 1) for the phylogenetic analysis were retrieved by BLASTP from the NCBI database or by TBLASTN from *L. brevidens* and *L. subracemosa* transcriptomic databanks (VanBuren et al. 2018) with the CpWAK1 protein sequence as query (*E* < 10^–10^). The top two hits of selected species were used for further analysis. The phylogenetic tree was constructed using the neighbor-joining method with 1000 bootstrap replications in MEGA 5.1 (Tamura et al. 2011). The genomic sequences for the gene structure analysis were obtained from NCBI database or identified using BLAST in *L. brevidens* and *L. subracemosa* genomic databanks (VanBuren et al. 2018). The gene structures of all selected WAK genes are displayed using Gene Structure Display Server 2.0 (http://gsds.cbi.pku.edu.cn/) (Hu et al. 2015).

**Table 1:** Selected homologs of CpWAK1

Accession number	Organism	Query coverage	Percent identity	*E *value
Lbr_010788	*Lindernia brevidens*	99%	69.73%	0.0
Lbr_015766	*Lindernia brevidens*	88%	53.20%	0.0
Lsu_027987	*Lindernia subracemosa*	97%	69.60%	0.0
Lsu_004124	*Lindernia subracemosa*	96%	61.30%	0.0
RXH67760.1	*Malus domestica* (apple)	94%	44.85%	0.0
XP_008380329.1	*Malus domestica* (apple)	95%	44.44%	0.0
NP_00132332.1	*Arabidopsis thaliana*	95%	43.20%	6e-174
NP_001185009.1	*Arabidopsis thaliana*	95%	43.06%	3e-175
EAZ21467.1	*Oryza sativa* (rice)	91%	38.14%	1e-134
XP_015627146.1	*Oryza sativa* (rice)	94%	37.73%	2e-135
XP_024376490.1	*Physcomitrella patens*	51%	43.34%	2e-84
XP_024376488.1	*Physcomitrella patens*	51%	43.34%	3e-84

Putative CpWAK homologs identified by BLASTP from the non-redundant protein sequences NCBI database (nr) or by TBLASTN from *L. brevidens* and *L. subracemosa* transcriptomic databanks (VanBuren et al. 2018). The first two hits of selected species were used for further phylogenetic analysis

The nucleotide sequences of the *C. plantagineum* genes described in this study are deposited in the NCBI GenBank database under the following accession numbers: KT893872 (CpWAK1), KT893873 (CpWAK2), MW580911 (CpWAK3), and KT893871 (CpGRP1).

### Statistical analysis

All the experiments were conducted using three biological replicates. The mean and the standard error of mean (SEM) values shown in the ELISA binding assays were calculated from three biological replicates including three technical replicates each (*n* = 9). Statistical significance was determined by t-test in Fig. 3b, c and Fig. 4a–d and by one-way ANOVA with Bonferroni's post-test in Figs. 3d and Fig. 4e. All statistical analyses were performed with Excel and Graphpad prism 5.0 (San Diego, CA; https://www.graph pad.com/).

## Results

### Phylogenetic analysis of CpWAK genes

This study is focused on three very closely related *CpWAK* genes isolated from the desiccation-tolerant plant *C. plantagineum*: *CpWAK1*, *2* and *3*. The deduced amino acid sequences of the three *CpWAK* genes show the high sequence conservation of the three predicted proteins (Fig. S2). All three CpWAK proteins display the features of wall-associated kinases: the extracellular galacturonide-binding domain, EGF repeats, a transmembrane domain and a cytoplasmic protein kinase domain (Fig. S2).

**Fig. 1:**
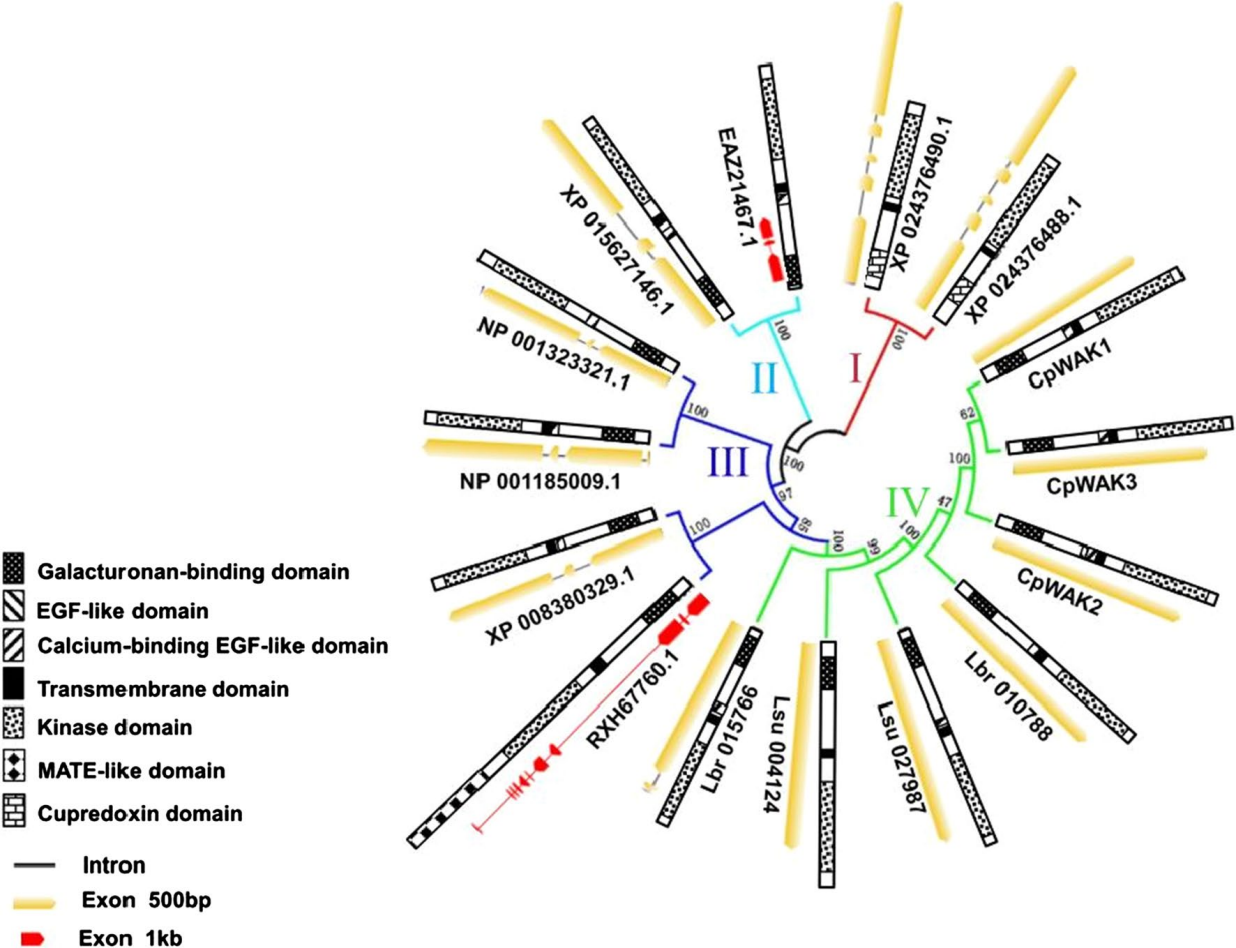
Phylogenetic analysis of WAK proteins. The WAK homologs for the phylogenetic analysis were retrieved by BLASTP from the NCBI database or by TBLASTN from *L. brevidens* and *L. subracemosa* transcriptomic databanks (VanBuren et al. 2018) with the CpWAK1 protein sequence as query (*E* < 10^–10^). The top two hits of selected species were used for further analysis. The 15 predicted WAK protein sequences of *Craterostigma plantagineum* (CpWAK1, 2 and 3), *Lindernia subracemosa* (Lsu_027987, Lsu_004124), *Lindernia brevidens* (Lbr_010788, Lbr_015766)*, Arabidopsis thaliana* (NP_001185009.1, NP_001323321.1), *Malus domestica* (RXH67760.1, XP_008380329.1), *Oryza sativa* (XP_015627146.1, EAZ21467.1) and *Physcomitrella patens* (XP_024376490.1, XP_024376488.1) were subjected to a multiple sequence alignment using the ClustalW program of MEGA 5.1 software (Tamura et al. 2011). The phylogenetic tree was then constructed by neighbor-joining method with 1000 bootstrap replications in MEGA 5.1 (Tamura et al. 2011). The bootstrap values displayed on branches indicate the reliable level to the nods of the tree. All homologs were divided into four clusters (group I: moss, group II: monocots, group III: dicots, group IV: Linderniaceae family, marked with different colors). The genomic sequences for the gene structure analysis were obtained from NCBI database or identified using BLAST in *L. brevidens* and *L. subracemosa* genomic databanks (VanBuren et al. 2018). The gene structures were generated using the Gene Structure Display Server 2.0 (http://gsds.cbi.pku.edu.cn/) (Hu et al. 2015). Exons in the gene structures are illustrated with red or yellow bars due to the different length of genes

To investigate the evolutionary relationships of the *CpWAK* genes with other species, a phylogenetic analysis was carried out of *WAK* genes from *C. plantagineum*, the moss *Physcomitrella patens* and selected vascular plants including two Linderniaceae species closely related to *C. plantagineum* (Fig. 1). The WAK homologs used for the phylogenetic analysis are reported in Table 1. As shown in Fig. 1 homologs are divided into four clusters which reflect the genus classification (group I: moss, group II: monocots, group III: dicots, group IV: Linderniaceae family). Only the WAK genes in group IV have no introns or fewer introns than the other genes (Fig. 1). The protein structures of all WAK homologs are highly similar, especially in vascular plants. All WAK proteins from vascular plants contain the conserved kinase domain, the EGF-like domain and the extracellular galacturonan-binding domain, while a MATE-like domain (cd13132) is present in RXH_67760.1 (*Malus domestica*) (Fig. 1). The MATE-like domain is related to iron homeostasis under osmotic stress. No transmembrane domain is predicted in NP_001323321.1 (AT1G16260, WAKL8, *A. thaliana*). The predicted WAK proteins of *P. patens* show similarity only within the conserved kinase domain and contain a cupredoxin motif (cl19115) located in the extracellular domain.

### Expression analyses of CpWAKs

**Fig. 2:**
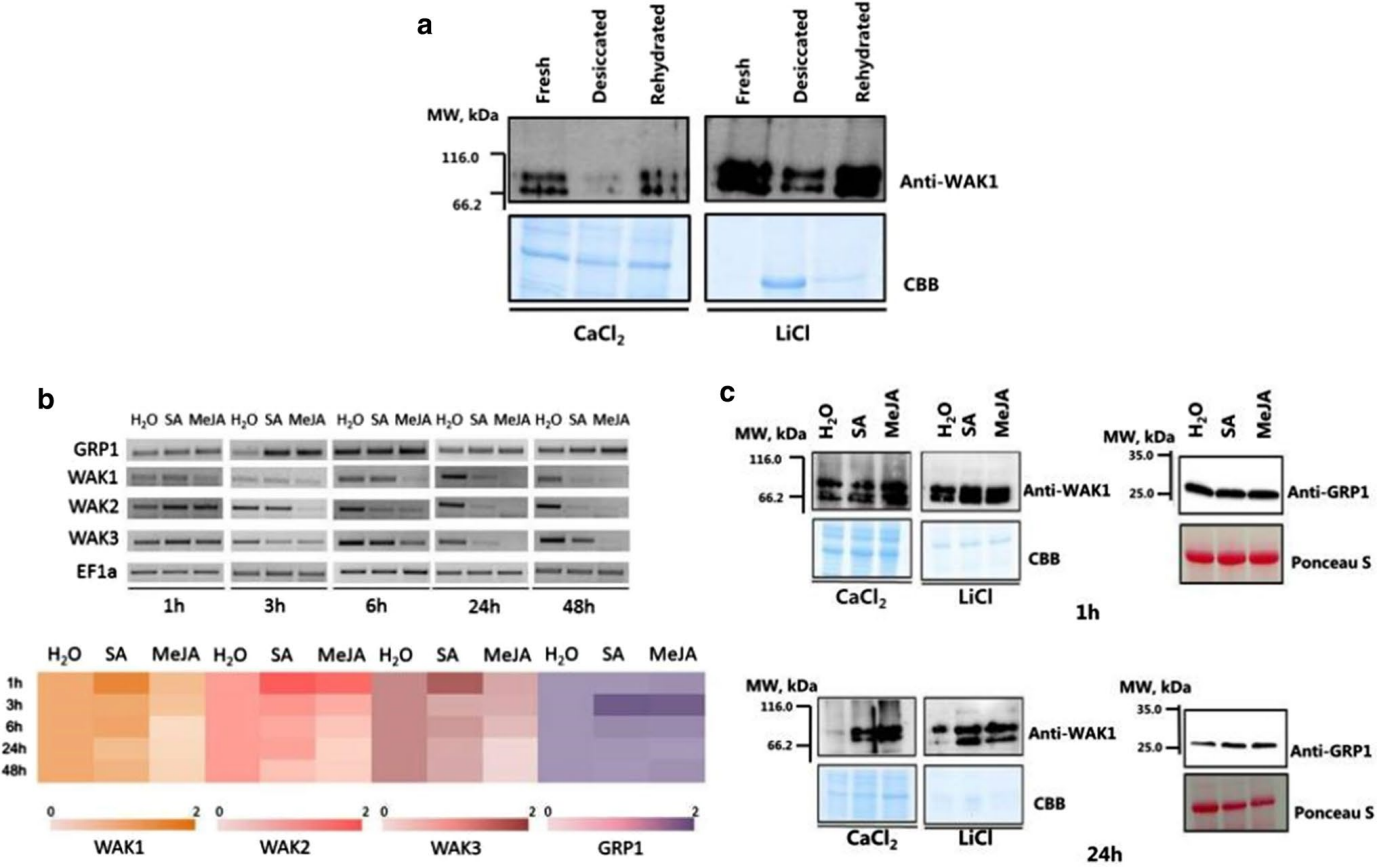
Transcript and protein expression analysis of *C. plantagineum* cell wall-associated protein kinases (CpWAKs) and its interacting partner *C. plantagineum* glycine-rich protein 1 (CpGRP1). **a** Protein expression pattern of CpWAKs under drought was analyzed using leaf samples collected from intact plants of *C. plantagineum* untreated (Fresh), dried in pots for 7 days (Desiccated), or rehydrated in pots for 3 days (Rehydrated). **b**, **c** Transcript and protein expression patterns of CpWAKs and CpGRP1 under salicylic acid (SA) or methyl jasmonate (MeJA) treatments. *C. plantagineum* detached leaves were exposed to 1 mM SA and 100 µM MeJA separately for 1, 3, 6, 24 and 48 h, while the leaves soaked in water served as control. The heatmap in b showed the semi-quantitative RT-PCR analysis of CpWAKs and CpGRP1 using ImageJ software. The relative expression levels of CpWAKs and CpGRP1 in SA and MeJA were normalized to that in water. *n* = 9. All protein samples were analyzed by 12% SDS-PAGE, transferred to nitrocellulose membrane, and detected by polyclonal antibodies (anti-CpWAK1 antibody 1:5000, anti-CpGRP1 antibody 1:5000). Protein expression analysis of CpWAKs and CpGRP1 were investigated in cell wall proteins and total proteins with CBB (Coomassie brilliant blue-stained) gels and Ponceau S-stained membranes as loading controls, respectively

It was previously shown that *CpWAK1* and *2* genes are mainly expressed in well-watered leaves of *C. plantagineum* and repressed by dehydration (Giarola et al. 2016). This suggests that expression of these two genes is associated with the rehydration process. To extend our understanding of the processes involving *CpWAK* genes we analyzed the expression of the three *CpWAK* genes using gene specific probes and a polyclonal antiserum. A polyclonal antiserum was raised against a recombinant fragment of CpWAK1. However, the antiserum detects probably also CpWAK2 and CpWAK3 proteins as well as other CpWAKs with conserved extracellular domains due to the high conservation of the amino acid sequences. The protein expression during dehydration/rehydration of CpWAKs is illustrated in Fig. 2a. The two protein bands on the blot may represent not only the CpWAK1 protein but other CpWAK proteins, or some posttranslationally modified WAKs.

WAK proteins are associated with cell wall compartments (Giarola et al. 2016) and were not detected in total protein extracts. Therefore, cell wall protein fractions were prepared according to the protocol 3 of Printz et al. (2015) and two different fractions (CaCl_2_ and LiCl fractions) were used for protein detection in immunoblots (Fig. 2a). In both fractions the CpWAK proteins are present in extracts of well-watered and rehydrated leaves and decreased during dehydration (Fig. 2a). Thus, the protein accumulation profile is similar to what was previously observed for transcripts (Giarola et al. 2016) suggesting that WAK gene expression is mainly regulated on the transcriptional level during dehydration/rehydration.

WAK proteins interact with GRP proteins in *C. plantagineum* and *A. thaliana* (Park et al. 2001; Giarola et al. 2016). In *Arabidopsis*, both WAK and GRP are up-regulated upon SA or the SA analog 2, 6-dichoroisonicotinic acid (INA) treatment (He et al. 1999; Park et al. 2001; Verica et al. 2003), which suggests that the two proteins are involved in defense-response pathways. Thus, the transcript and protein expression of the CpWAK genes and CpGRP1 were analyzed in *C. plantagineum* upon SA and MeJA treatments (Fig. 2b, c). *CpWAK1, 2 and 3* show a faster response than CpGRP1 and they accumulate after 1 h of SA treatment, while *CpGRP1* accumulated after 3 h of SA and MeJA treatments (Fig. 2b). CpWAK1 and CpWAK3 decrease in response to MeJA, while CpWAK2 increases (Fig. 2b). The accumulation of all transcripts is transient and the levels decrease quickly after reaching the peak. No significant difference was observed in the expression of CpWAKs and CpGRP1 proteins after 1 h of SA and MeJA treatments, but CpWAKs and CpGRP1 proteins accumulate after 24 h of treatments (Fig. 2c). The expression of CpWAKs and CpGRP1 proteins is still detectable in the water-treated samples even up to 24 h, which is in line with the transcript expression pattern (Fig. 2b, c). The transcript and protein expression patterns point to a different regulation in response to SA or MeJA than in response to dehydration. The expression analysis suggests that CpWAK and CpGRP1 proteins are involved in defense-response pathways possibly triggered in the cell wall.

### CpWAKs form aggregates and have a high affinity to the pectins in cell wall “egg-box” conformations

**Fig. 3:**
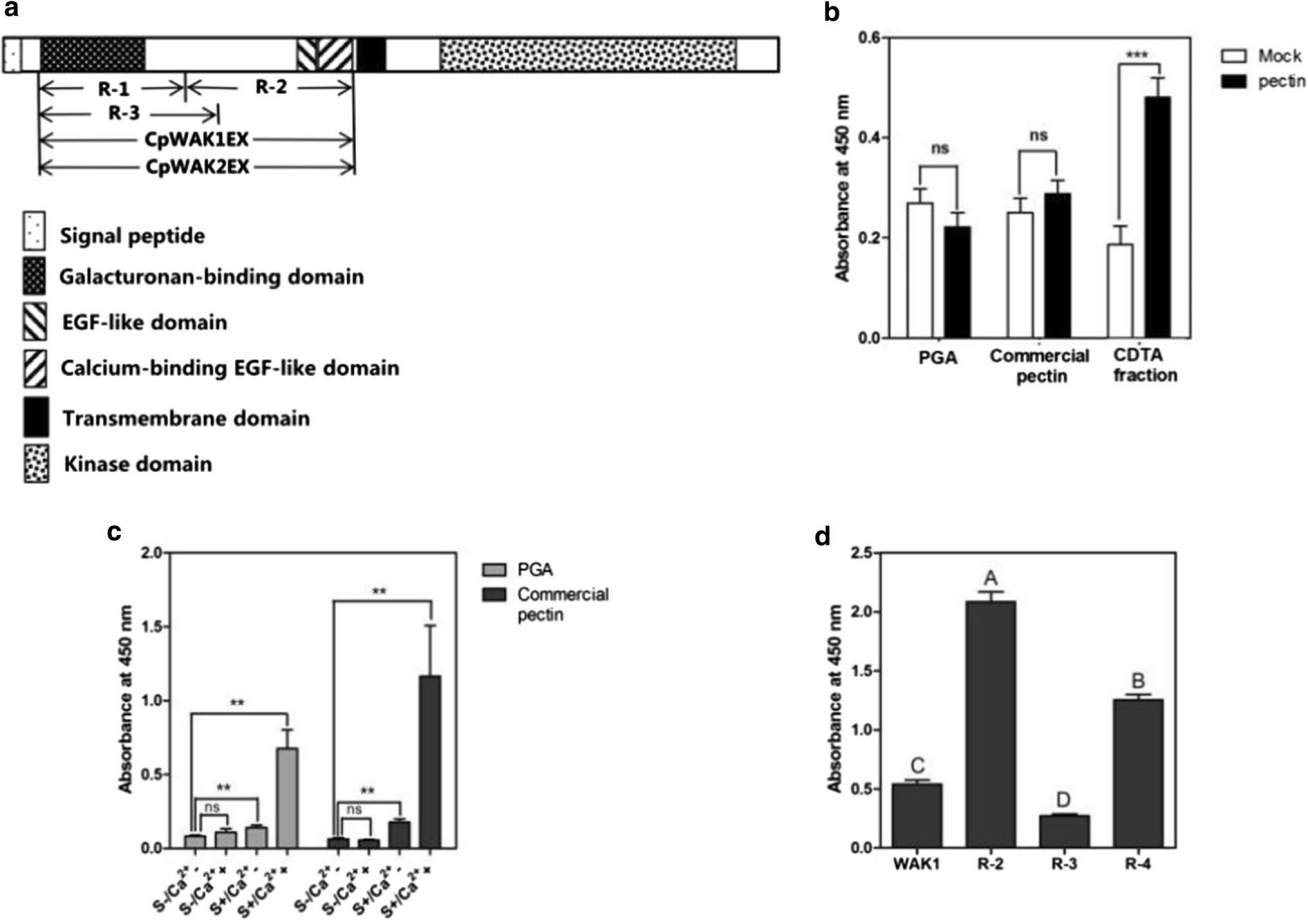
CpWAKs show higher affinity to the pectins in “egg-box” conformation. **a** Domain structures of CpWAK proteins and the fragments for His-tagged recombinant proteins. The extracellular domains of CpWAK proteins without signal peptides are CpWAK1EX (amino acid 31–315) and CpWAK2EX (37–333 for CpWAK2EX). R-1, R-2 and R-3 are truncated fragments of the CpWAK1EX protein, containing the amino acids 31–160, 161–315 and 31–220, respectively. **b** The CpWAK1 extracellular domain only binds to the pectin extracts of *C. plantagineum* leaves. **c** Both saponification and Ca^2+^ are necessary for higher binding capacity of the CpWAK1 extracellular domain to polygalacturonic acid and commercial pectin. S ± : saponificated/non-saponificated pectins; Ca^2+^  ± : buffer with or without Ca^2+^. **d** Different subdomains of CpWAK1 showed different pectin binding capacity. WAK1EX, WAK2EX, R-1, R-2 and R-3 represent different fragments as shown in **a**. Polygalacturonic acid (PGA, Sigma), commercial pectin (pectin from citrus peel, Sigma) and *C. plantagineum* pectin isolated from *C. plantagineum* leaves with CDTA (1, 2-cyclohexanediaminetetraacetic acid) solution were normalized by pectin estimation assay, and immobilized in ELISA plate wells, incubated with 0.2 µg of purified recombinant CpWAK1EX or the same amount of other recombinant proteins. The bound recombinant proteins were detected with His-tag antibody (1:10,000). All the mean absorbance values were calculated from three biological of which each included three technical repetitions. Error bars indicate SEM and Mock indicates only buffer without pectin (ns means no significant, ***P < 0.0001, **P < 0.01, *t *test compared to Mock in **b** and S-/Ca^2+^- in **c,** respectively). The letters in **d** show the significance determined by one-way ANOVA with Bonferroni’s post-test (**a**–**d** *P* < 0.01)

To better understand how WAK proteins can mediate signalling we overexpressed different CpWAK protein fragments in bacteria and used these fragments for binding studies (Fig. 3a). Here, CpWAK1EX and CpWAK2EX are used to represent the extracellular domains of CpWAK proteins without signal peptides; whereas, R-1, R-2 and R-3 are truncated fragments of the CpWAK1 protein, containing the amino acids 31–160, 161–315 and 31–220, respectively (Fig. 3a, Fig. S3). The putative molecular weight and pI of the His-tagged recombinant proteins are reported in Table 2.

**Table 2:** Basic characteristics of the His-tag recombinant proteins

Recombinant proteins	Amino acids covered	Molecular weight (kDa)	Isoelectric point (pI)
CpWAK1EX	31–315	32.2	4.71
CpWAK2EX	37–333	34.1	5.31
R-1	31–160	14.9	7.74
R-2	161–315	18.6	4.31
R-3	31–220	21.8	6.22
CpGRP1	22–156	14.18	8.35

Western-blot analyses of the CpWAK recombinant proteins confirmed that all proteins can be immunologically detected with anti-His-tag or with anti-CpWAK1 antisera (Fig. S3). The immunoblots show that the CpWAK recombinant proteins form multimers, especially R-1 (Fig. S3). Protein aggregation was facilitated by the addition of Ca^2+^ as protein bands were only detected in the bottom layer of SDS-PAGE gels in the samples containing CpWAK1 extracellular protein fragments supplemented with 2 mM Ca^2+^ (Fig. S4).

**Fig. 4:**
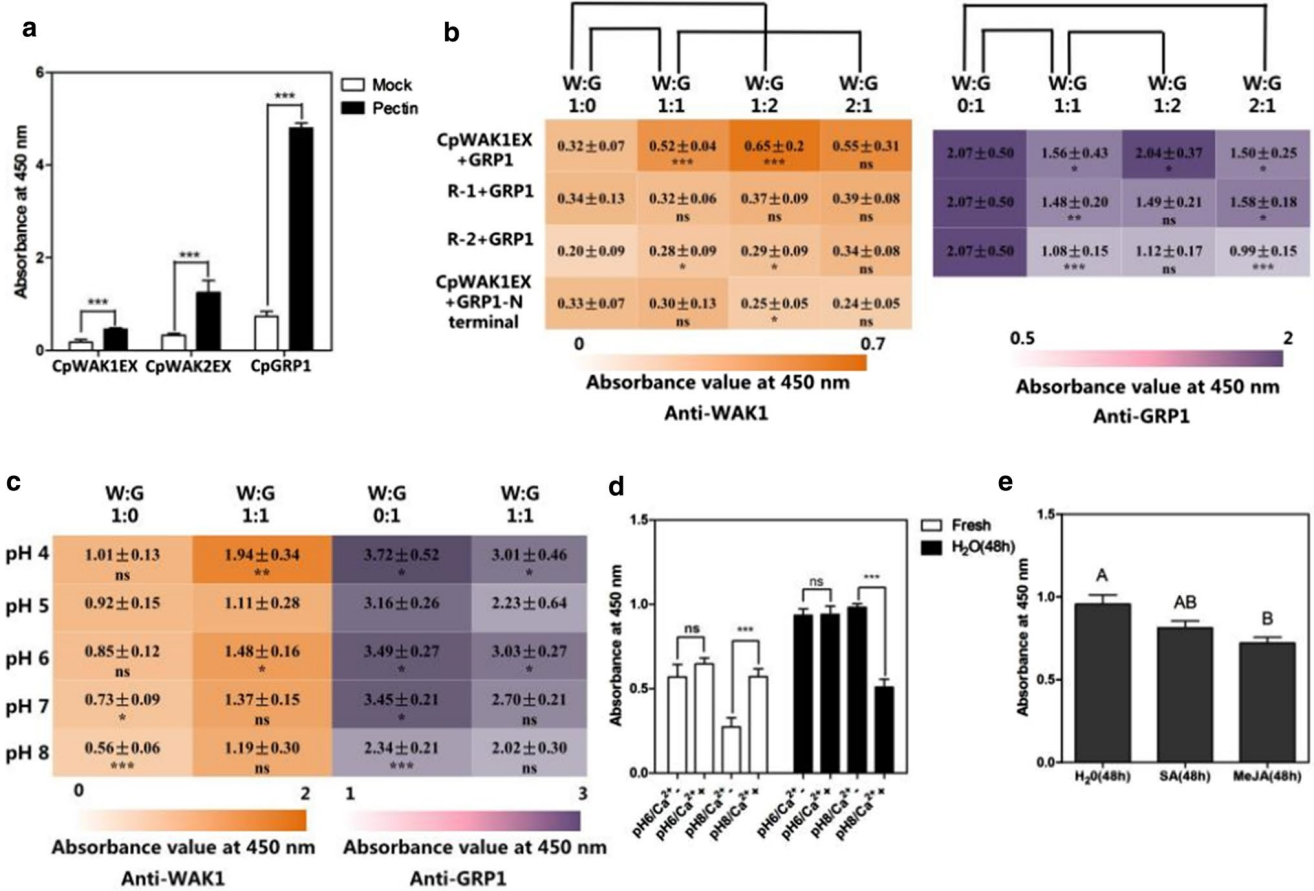
CpGRP1–CpWAK1–pectin interaction and the factors involved in CpWAK1–pectin binding*.* **a** The interaction of the pectin extracts of *C. plantagineum* leaves with CpGRP1 is much stronger than with CpWAKs. **b** Heatmaps showing the contribution of CpGRP1 to the binding activity of CpWAK1EX to pectins. **c** CpWAK1EX–CpGRP1 complex binding affinity for pectin extracts is affected by pH values. Each heatmap depicts the ELISA absorbance values with anti-CpWAK1 (orange, 1:2500) and anti-CpGRP1 (purple, 1:5000) antibody, respectively. Values shown in the heatmaps are means of three biological replicates which included three technical replicates ± SD. The lines above the heatmap in **b** are the combinations for *t *test. Different protein combinations or different pH values are visualized longitudinally, and the ratios of the amounts of CpWAKs (W) and CpGRPs (G) are shown horizontally. The proteins were pre-mixed for 1 h before incubating with the pectin-immobilized ELISA plates. **d** The effect of Ca^2+^ on the CpWAK1EX–pectins binding is related to pH values and the source of pectin extracts. Pectins were extracted from untreated *C. plantagineum* leaves (Fresh) and *C. plantagineum* leaves incubated in water (H_2_O). The treated leaves were soaked for 48 h. Asterisks in the heatmaps and above bars represent statistically significant differences compared to control samples (Mock in **a**, W:G = 1:0 or W:G = 1:1 in **b**, pH 5 in **c,** Ca^2+^- in **d**) (t-test, ns means no significant, *P < 0.05, **P < 0.01, ***P < 0.0001). **e** CpWAK1EX showed different binding capacity to the pectin extracts prepared from detached *C. plantagineum* leaves exposed to water, SA or MeJA for 48 h. Statistical analysis was performed using one-way ANOVA with Bonferroni’s post-test (**a**, **b**, *P* < 0.01); *n* = 9. Error bars indicate SEM

The extracellular domain of the AtWAK1 binds to pectins (Decreux and Messiaen 2005); therefore, the five CpWAK protein fragments were tested for interaction with either commercial pectin (poly-d-galacturonic acid methyl ester and galacturonic acid content ≥ 74%, Sigma Aldrich, P9135) from citrus peel or pectin isolated from *C. plantagineum.* CpWAK1EX–pectin binding was observed with the pectin extracted from *C. plantagineum* leaves, but not with commercial pectins or polygalacturonic acid (PGA, Sigma 81325) (Fig. 3b). Binding of CpWAK1EX to commercial pectin was achieved after saponification with and without Ca^2+^ (Fig. 3c). Saponificaiton and Ca^2+^ facilitate the formation of “egg-box” structures of pectins (Grant et al. 1973; Cabrera et al. 2008). CpWAK1EX showed a higher preference to the pectins in “egg-box” conformation (supplemented with Ca^2+^) than those only saponificated but without Ca^2+^ (Fig. 3b). Like CpWAK1EX, CpWAK2EX also showed affinity for pectins from *C. plantagineum* and Ca^2+^ facilitates the binding of the two extracellular domain of the CpWAKs to pectin extracts (Fig. 4a, S5a). Next, we tested the affinity of R-1, R-2, and R-3 fragments to pectins. R-1 includes the galacturonan-binding domain, R-2 contains the EGF-like domains and R-3 corresponds to R-1 but includes alkaline amino acids more towards the 3`end without the EGF-like domains (Fig. 3a). Among the three fragments, R-1 showed the strongest binding capacity to *C. plantagineum* pectin extracts, closely followed by R-3. R-2, containing no galacturonic binding domain, had less affinity than other fragments (Fig. 3d).

### The CpWAK1–pectin–CpGRP1 complex

CpWAK1 does not only interact with pectins but also with the glycine-rich cell wall protein CpGRP1 (Giarola et al. 2016). CpGRP1 has a stronger affinity to pectins than CpWAKs (Jung et al. 2019) (Fig. 4a). Therefore, the interaction of CpWAK1 and pectin was further tested including the CpGRP1 protein using competitive ELISA assays. Equal molar amounts of CpWAK1EX and CpGRP1 proteins were premixed before incubating them with pectin. The heatmaps in Fig. 4b show that full-size CpGRP1 contributes to significantly more binding of CpWAK1EX and R-2 to pectin extracts. However, the presence of CpWAK1 decreases the number of CpGRP1 molecules binding to pectin extracts, especially in experiments with the R-2 fragment, which might contain the site for the interaction with CpGRP1. The N-terminal fragment of CpGRP1 without the pectin-binding domain (Jung et al. 2019) significantly decreases CpWAK1EX–pectin binding when the ratio of WAK/GRP is 1:2 (Fig. 4b).

The apoplastic pH is normally around five and oscillates between 4 and 7, which is observed in specific developmental stage or under stress conditions (Geilfus 2017). Thus, we investigated the effect of the apoplastic pH on the CpWAK1/CpGRP1/pectin interaction varying the pH of ELISA binding assays (Fig. 4c). The binding between CpWAK1EX and pectins was not significantly affected over the pH range of 4–7, but with increasing pH, a decrease was observed. However, CpGRP1 showed stronger binding capacity at pH 4, 6 and 7. When CpGRP1 and CpWAK1EX proteins were mixed and the pectin binding was tested at different pH values, the same trend was seen in all assays as for CpGRP1 alone despite the insignificant difference in pH 7. Ca^2+^ affects CpWAK1EX–pectin binding depending on the pH and the source of the pectin, only when the pH value increased to 8.0 (Fig. 4d). The binding was strengthened when the pectin was extracted from untreated leaves, while weakened when pectin was prepared from water-soaked leaves. The SA and MeJA treatments also affected the CpWAK1EX–pectin binding, with less CpWAK1EX being immobilized by pectin extracts from detached leaves subjected to SA and MeJA treatments (Fig. 4e). However, CpWAK1EX did not show significantly different affinity for the pectin extracted from untreated, partially dried, desiccated and rehydrated *C. plantagineum* leaves (Fig. S5b**)**.

## Discussion

### Evolution of CpWAKs

The genome-wide analyses of WAKs in rice, *Arabidopsis* and apple indicated that tandem duplication and segmental duplications contribute to the expansion of WAK gene families (Shiu et al. 2004; Zhang et al. 2005; Zuo et al. 2019). The high sequence similarity among the CpWAKs (Fig. S2) suggests also duplications for the *CpWAK* genes in *C. plantagineum*. The phylogenetic analysis shows that the WAK homologs from different species are clustered in distinct species-specific groups (Fig. 1), which is consistent with that in rice, *Arabidopsis* and apple (Zhang et al. 2005; Zuo et al. 2019). This suggests that gene expansion/gene duplication takes place after species divergence. It is remarkable that a non-intron gene structure is only seen in the Linderniaceae family (group IV) among the selected *WAK* gene homologs (Fig. 1). The amino acid sequences of the selected WAKs are conserved within the cytoplasmic kinase domain, the EGF-like domain and the galacturonan-binding domains, but they are variable within the extracellular domains. This variability in the extracellular domain may be connected with a differential response of the *WAK* genes to environmental stimuli (Fig. 1). The extracellular domains exert special functions in some biological processes, such as the copper-binding-like domain (Cupredoxin domain) in XP_024376490.1 and XP_024376488.1 (Fig. 1). The conserved kinase domains of plant WAKs have also evolved into two classes: WAK-RD and WAK-non-RD after the monocot–dicot separation (de Oliveira et al. 2014). The classification of RD and non-RD classes depends on the presence of a conserved arginine (R) residue before the catalytic motif DxxxxN. All the three CpWAKs possess the RDxxxxN motif (Fig. S2) and, thus, belong to the WAK-RD class. The non-RD WAKs only occur in monocots. The different catalytic domains in the two WAK classes may lead to different signaling pathways (Kohorn 2015). The variability within the extracellular domain and the kinase domain may determine the specificity of WAKs in different biological processes.

### The expression patterns of CpWAKs

*WAK* gene expression is modulated by diverse stimuli and differential expression patterns have been observed on the transcript and protein level in *C. plantagineum*. For example*,* the transcript expression of *CpWAK*s was only slightly up-regulated after 1 h of SA treatment and reduced after 24 h treatment (Fig. 2b). The transcripts of *CpWAK1* and *CpWAK3* were suppressed under MeJA treatment while *CpWAK2* accumulated after 1 h MeJA treatment and then sharply declined (Fig. 2b**)**. CpWAK proteins accumulated after 6 h and 24 h of SA and MeJA treatments while no significant up-regulated expression patterns were observed on the transcript level (Fig. 2b,c**)**. This means that the expression of CpWAKs under SA and MeJA are mainly post transcriptionally controlled. In contrast to the modulation of CpWAK expression under SA and MeJA treatment, the expression of *CpWAK*s genes is regulated on the transcriptional level in response to dehydration, because the expression of CpWAKs showed the same trend on the transcript and protein level (Giarola et al. 2016) (Fig. 2a). Therefore, CpWAK expression may be controlled depending on the stimulus.

### CpWAKs form multimers

**Fig. 5:**
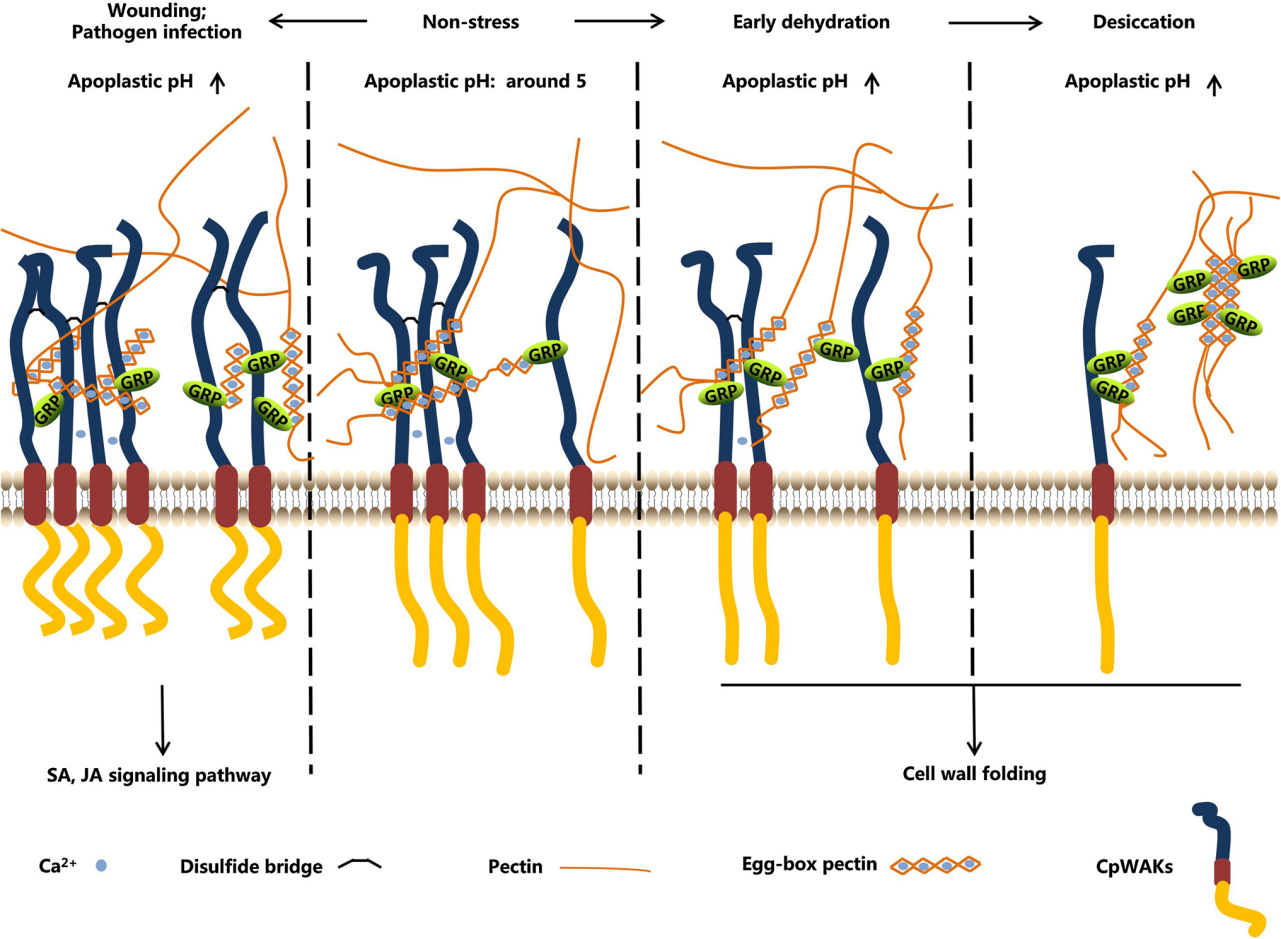
A model proposed for the activity of CpWAK proteins. CpWAKs are a group of cell wall-located receptor protein kinases are suggested to mediate responses to dehydration and defence to wounding or pathogen infections. CpGRPs are interaction partners of CpWAKs in the cell wall matrix. Both CpWAKs and CpGRPs interact with pectins preferentially in the “egg-box” conformation. The binding capacity of CpGRPs to pectins is sensitive to pH changes. In the early stage of dehydration, CpGRPs are up-regulated while less CpWAKs are accumulated. The linkages of CpWAKs to pectins can be strengthened indirectly by an increase of the apoplastic pH (pH_apo_) with the aid of CpGRPs. Under desiccation, a considerable amount of CpWAKs are degraded, which may result in less CpWAK multimers or dimers. Although the increased amount of CpGRPs contribute to the binding of CpWAKs to pectins, the decreased multimers or dimers may become the major factor negatively affecting the binding of CpWAKs to pectins. In contrast to the early stage of dehydration, wounding and pathogen infections induce accumulation of CpWAKs and cell wall pectin fragmentation whereas similarly to dehydration the pH_apo_ and accumulation of CpGRPs increase. Accumulation of CpWAKs results in the formation of more multimers or dimers, thus facilitating the binding of CpWAKs to pectins. The pectin fragments have a higher affinity for CpWAKs than the long pectin oligogalacturonides. The combined effects of the pH_apo,_ the modulation of CpGRPs, the formation of CpWAK multimers and the state of cell wall pectins may determine the specificities of the responses

Western blot analyses of purified recombinant proteins reveal two or more bands with His-tag or CpWAK1 antiserum (Fig. S3). The multiple bands suggest that CpWAKs can form dimers or multimers. The formation of multimers is mainly due to the presence of a protein domain within the R-1 fragment because more bands are seen when only the R-1 fragment was run on the gel. It was noticed that except for protein fragment R-2 the proteins of truncated fragments of CpWAKs do not migrate into the SDS-PAGE gels without DTT (Fig. S6). There are several cysteine residues localized in the galacturonan-binding domains, in the EGF-like domains and other parts of the CpWAK proteins (Fig. S2). These cysteine residues, especially those in the R-1 segment, are presumably responsible for the formation of CpWAK dimers or multimers via intermolecular disulfide bonds (Fig. 5). Besides the cysteine-rich domains, the EGF repeats can also lead to the dimerization of proteins mediated by calcium (Anderson et al. 2001; Verica et al. 2003). The recombinant protein corresponding to the extracellular region of CpWAK1 precipitates in the presence of Ca^2+^ (Fig. S4), which may result from the calcium-mediated protein dimerization of the EGF repeats.

### CpWAKs bind to pectins

It had been suggested that CpWAK1 is involved in the reversible folding of the cell wall during dehydration and rehydration in *C. plantagineum* (Giarola et al. 2016). Therefore, the underlying mechanisms of CpWAK-mediated cell wall folding were analyzed in detail.

Decreux and Messiaen (2005) first identified the WAK–pectin binding in vitro. WAK–pectin binding was also observed using the recombinant proteins of CpWAKs and the pectin extracts from untreated *C. plantagineum* (Fig. 3b) (Jung et al. 2019). According to Decreux and Messiaen (2005), AtWAK1 shows higher affinity for the pectins in the “egg-box” model. This is consistent with the observation for *C. plantagineum* (Fig. 3c, S5a). CpWAK1EX did not bind to polygalacturonic acid (PGA, Sigma) or commercial pectin (pectin from citrus peel, Sigma) (Fig. 3b), but CpWAK1EX–pectin binding was observed after saponification of commercial pectin in the presence of Ca^2+^ (Fig. 3c). Saponification breaks ester bonds and then the pectic chains can form the “egg-box” structure in a calcium environment (Sedan et al. 2007). CpWAK1EX showed a weak binding to saponificated pectins in buffer without Ca^2+^ (Fig, 3c), which indicates that CpWAK1 can bind to the negatively charged pectins. Fully de-methylesterified homogalacturonan was detected in the pectin extracts (CDTA fraction) from untreated *C. plantagineum* leaves (Jung et al. 2019). This is the prerequisite for the formation of “egg-box” structures in a calcium environment, which also explains the stronger binding between CpWAK1EX and *C. plantagineum* pectin. The binding assays using different fragments of the CpWAK1 proteins showed that both R-1 and R-3 fragments containing the galacturonan-binding domain bound more strongly to pectin than the R-2 fragment containing only EGF repeats (Fig. 3a, d). This demonstrates the importance of the galacturonan-binding domain in the CpWAK–pectin interaction. Protein band shift assays with CpWAK protein and pectin did not show a band shift on PAGE gels in the presence of DTT (Fig. S7). Therefore, the cleavage of the disulfide bonds with DTT not only disrupts the formation of multimers but also prevents the binding of CpWAKs to pectins. This suggests that CpWAKs bind to pectins as dimers or multimers via disulfide bonds (Fig. 5), consistent with the proposal that the extracellular domain of WAK proteins may function in a carbohydrate-rich environment involving protein interactions or oligomerization (He et al. 1999).

### CpGRP1 contributes to the binding of CpWAK1 to pectins

CpWAK1 does not only bind pectins, but it also forms a complex with the cell wall protein CpGRP1 (Giarola et al. 2016), which in turn influences the CpWAK1–pectin interaction. The heatmap in Fig. 4b shows that more CpGRP1 molecules lead to more binding of CpWAK1EX to pectins but in contrast more CpWAK1EX molecules lead to less CpGRP1 binding. The N-terminal fragment of CpGRP1 without the pectin-binding domain competes with pectins for interacting with CpWAK1EX (Fig. 4b). These results indicate that CpGRP1 contributes to the binding of CpWAK1EX to pectins by interacting with CpWAK1EX (Fig. 5) which supports the hypothesis proposed by Giarola et al. (2016) that CpWAK1 interactions with cell wall components are modulated by additional interaction partners such as CpGRP1. GRPs interact with the extracellular domain of WAKs (Park et al. 2001; Giarola et al. 2016). The effect of CpGRP1 on the binding of CpWAK1 and pectins was also observed using the CpWAK1 fragments, R-1 and R-2 (Fig. 4b). The R-2 fragment reduces the binding of CpGRP1 to pectins, which implies that CpGRP1 mainly interacts with a protein domain present within the R-2 fragment of the CpWAK1.

### Multiple factors influence CpWAK–pectin binding

CpWAK protein abundance decreases in response to dehydration (Fig. 2a). Based on the responses of CpWAKs and CpGRP1 to dehydration, Giarola et al (2016) proposed that the CpGRP1–CpWAK1 complex is implicated in the cell wall remodelling during dehydration and rehydration. The association of WAKs with wounding and pathogenesis-related processes is partially due to the increased expression after wounding or pathogen infection (Park et al. 2001; Kohorn and Kohorn 2012; Kohorn 2015). Plant hormones SA and JA are involved in the responses of plants to pathogens and wounding (Dong 1998; Reymond and Farmer 1998). The protein expression of CpWAKs can be induced by both SA and MeJA (Fig. 2c). Treatments of *C. plantagineum* leaves with the two hormones also resulted in increased expression of CpGRPs (Fig. 2c). The accumulation of CpWAK and CpGRP proteins seem to follow similar kinetics, with an increase after 6 h (Fig. 2c). The simultaneous accumulation of CpWAKs and CpGRPs make it possible that CpGRP acts as a modulator in regulating the cell wall signal perception of CpWAKs after wounding or pathogen infection. CpGRPs and CpWAKs show antagonism in the OG/flg22/wound-triggered defense responses according to Gramegna et al. (2016).

All pectin binding assays described above were performed in buffered solutions with pH 8. However, the pH value of the apoplast (pH_apo_) is generally around 5 and can also vary in the range from 4 to 7 depending on the physiological conditions (Grignon and Sentenac 1991). Acidification or alkalinization of the apoplast takes place in growing tissues and in tissues under stress (Grignon and Sentenac 1991; Geilfus 2017). The pectin-binding of CpWAK1EX per se did not show significant differences at pH values from 4 to 6, but the CpWAK1EX–CpGRP1 complex was sensitive to pH changes, showing stronger affinity for pectins at pH 4, 6 and 7 (Fig. 4c). The influence of CpGRP1 on the interaction of CpWAK1EX and pectins at different pH values means that CpGRP1 may be able to differentiate signals via perception of pH_apo_ changes. Thus CpWAK1–CpGRP1 cannot only be involved in cell wall loosening and cell expansion induced by the acidification of the apoplast but also in stress responses (Kohorn 2015; Giarola et al. 2016). Ca^2+^ enhances the binding between AtWAK1 and pectins (Decreux and Messiaen 2005). WAKs show higher affinity to pectins in calcium-induced “egg-box” structures formed with de-methylesterified pectins (Decreux and Messiaen 2005). This was also observed for CpWAKs, but only at pH 8 using the pectins isolated from untreated *C. plantagineum* leaves (Fig. 4d, S5a). The insignificant effect of Ca^2+^ on the WAK–pectin binding may arise from the saturation of the binding sites of the de-esterified pectins. The opposite effects of Ca^2+^ on the CpWAK1EX binding to different pectin extracts at pH 8 may result from the effect of the pH on CpWAK1EX conformation and/or different cell wall compositions in the pectin samples. Therefore, it is likely that CpGRP1 prioritize the Ca^2+^ in modulating the CpWAK1–pectin binding in non-stress conditions. Presumably Ca^2+^ influences the CpWAK1–pectin linkage and, thus, triggers specific signaling pathways under extreme conditions.

The de-methylesterified pectins in the cell wall are necessary for the formation of “egg-box” gelatin. The pectins can be de-methylesterified by pectin methylesterases, which is inhibited by the pectin methylesterase inhibitors (Micheli 2001). Previous studies showed that SA and MeJA led to the up-regulated expression of pectin methylesterase inhibitors and, thus, gave rise to the controlled activity of pectin methylesterases and decreased de-methylesterified pectins (An et al. 2008; Meng et al. 2009). Thus, the weaker binding of CpWAK1EX to pectin extracts from SA and MeJA-treated *C. plantagineum* leaves (Fig. 4e) may result from fewer “egg-box” structures in the cell wall pectins resulting from lower activity of pectin methylesterases.

Although more pectins in “egg-box” conformation may be present in desiccated tissues (Vicré et al. 2004; Jung et al. 2019), CpWAK1EX did not show different affinities to pectins extracted from hydrated or dehydrated *C. plantagineum* leaves (Fig. S5b). These observations imply that CpGRP1 may affect CpWAK–pectin binding during the early stages of dehydration (Fig. 5). During desiccation, the low abundance of CpWAKs leads to a decrease of WAK dimers and multimers which promote CpWAK–pectin binding; therefore, abundance of CpWAKs may distinguish the signals under desiccation (Fig. 5).

WAKs as wall-associated receptor kinases should be capable of recognizing different signals with the help of ions and protein ligands. It is a big challenge to understand how WAKs distinguish the different signals from cell walls. In this work, CpGRP1, pH_apo_, Ca^2+^_[apo]_ and the formation of CpWAK multimers or dimers are considered as potential factors involved in the orchestrated processes. Each factor has a potential to play a role in the regulation of signal perception under stress (Fig. 5). The hypothesis is that CpWAK decodes cell wall signals in concert with CpGRP1, which modulates CpWAK–pectin interaction in responses to wounding or pathogen infections, and a decrease of CpWAKs multimers may activate downstream signals during desiccation.

#### *Author contribution statement*

PC planned and designed the research, conducted the experiments and wrote the manuscript. DB and VG designed the research, supervised the work and corrected the manuscript.

## Supplementary Information

Below is the link to the electronic supplementary material.

Supplementary file1 (DOC 1413 KB)

## Data Availability

All data supporting the findings of this study are available within the manuscript and within its supplementary materials published online.

## Abbreviations

At: *Arabidopsis thaliana*
Cp: *Craterostigma plantagineum*
CDTA: 1, 2-Cyclohexanediaminetetraacetic acid
EGF: Epidermal growth factor
GRP: Glycine-rich protein
Lbr: *Lindernia brevidens*
Lsu: *Lindernia subracemosa*
MeJA: Methyl jasmonic acid
OG: Oligo-galacturonides
SA: Salicylic acid
WAK: Wall-associated protein kinase
WAKL: WAK-like kinase
