# Supplementary material for: The Craterostigma plantagineum protein kinase CpWAK1 interacts with pectin and integrates different environmental signals in the cell wall
Source: Planta. 2021 Apr 5;253(5):92. doi: 10.1007/s00425-021-03609-0 (PMC8021526; doi:10.1007/s00425-021-03609-0)
Supplement: Supplementary file 1 — Supplementary file1 (DOC 1413 KB) [file 425_2021_3609_MOESM1_ESM.doc]

**Supplementary data**

**Table S1.** List of primers used in this study.

| *Primers* | *Sequence (5’ 🡪3’)* | *Restriction site* |
| --- | --- | --- |
| CpWAK_F | CACGGAAATGAGGACTTCTTC |  |
| CpWAK1_F | AAATGAGGACTTCTTCAATACTTGCT |  |
| CpWAK_R | TCAATATCCATAACCGATAATCCA |  |
| pJET1.2_F  CpWAK1_XhoI_R  R-1 | CGACTCACTATAGGGAGAGCGGC  ATACTGCTCGAGACACGTGAAAGAGC  ATACTGCTCGAGCGTGTAATTCTTTCC | *XhoI*  *XhoI* |
| R-2-for | ACTACCATGGATCAAGACTCTTGCCA | *NcoI* |
| R-2-rev | ATACTGCTCGAGACACGTGAAAGAGC | *XhoI* |
| R-3 | ATACTGCTCGAGCAAATAATCATCGCT | *XhoI* |
| CpWAK2-XhoI-R | ATACTGCTCGAGACATGTGGAAGAG | *XhoI* |
| T7 promoter  T7 terminator  CpWAK1_RT_F | TAATACGACTCACTATAGGG  GCTAGTTATTGCTCAGCGG  GAGCTCAGAGTGCAGTATCGAC |  |
| CpWAK1_RT_R | GGGAAAGATCGCTAAATCGAG |  |
| CpWAK2_RT_F | AAGGTGCTGTGCAGACTCATC |  |
| CpWAK2_RT_R | AAGATTGCCCCAGTAATATGGAC |  |
| CpWAK3_RT_F | AGAGTGCAGTATCGGCTTACAT |  |
| CpWAK3_RT_R | CTCACGCTATATCGACTTGAGCA |  |
| CpGRP1_RT_F | GGTGGTTATGGTGGAAGAGG |  |
| CpGRP1_RT_R | TTGCATGAATGAAACGGAGAT |  |
| CpEF1 α_RT_F | AGTCAAGTCCGTCGAAATGC |  |
| CpEF1 α_RT_R | CACTTGGCACCCTTCTTAGC |  |


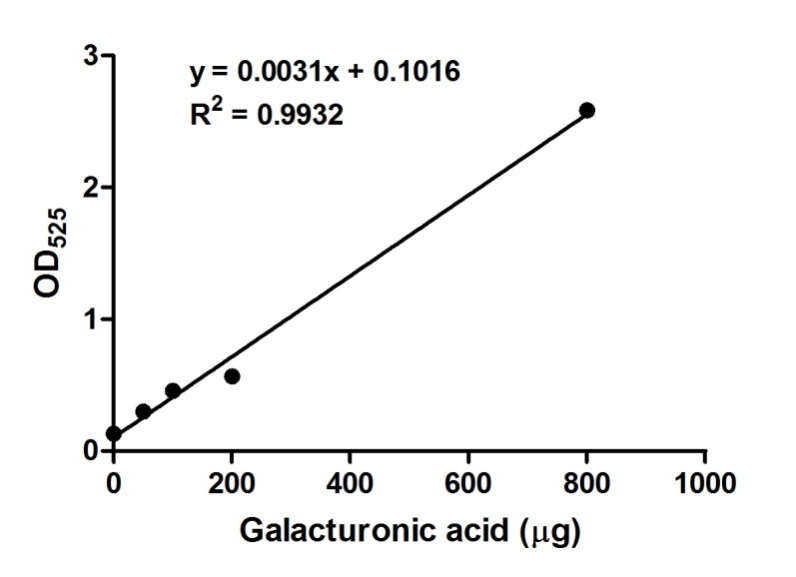


**Fig. S1.** Galacturonic acid standard curve for determining the galacturonic-acid content in pectin extractions.


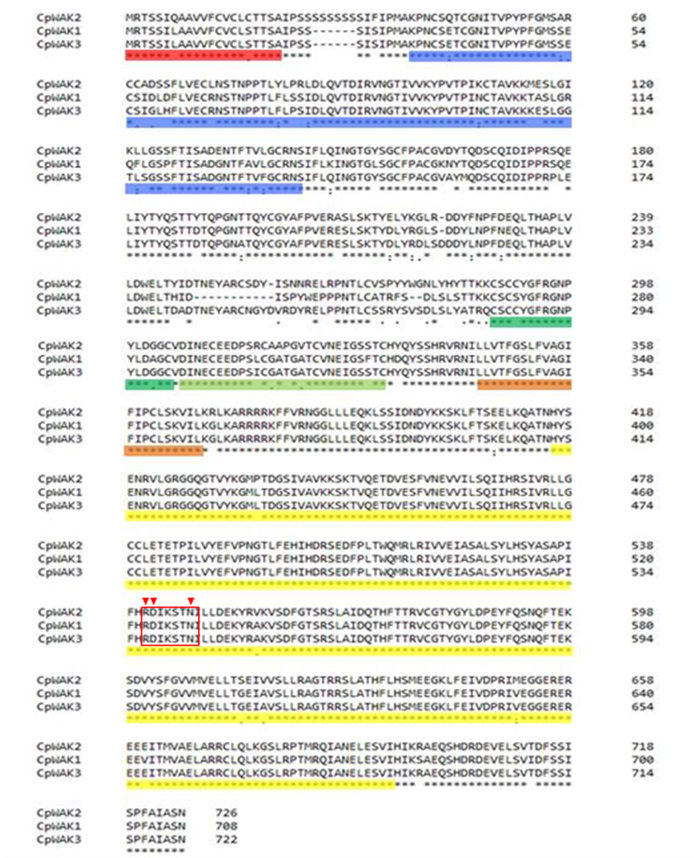


**Fig. S2**. Alignment of the predicted CpWAK1, CpWAK2 and CpWAK3 protein sequences (modified from Giarola et al. 2016). The alignment analysis of the three CpWAK protein sequences was performed using Clustal Omega (https://www.ebi.ac.uk/Tools/msa/clustalo/). The symbols in the figure indicate the fully conserved positions (asterisk “*”), conserved substitutions (colon “:”), and semi-conserved substitutions (period “.”), respectively. Protein domains were identified according to Giarola et al. (2016). Different coloured strips highlight different domains. Red: predicted signal peptide; blue: galacturonan-binding domain; dark green: EGF-like domain; light green: calcium-binding EGF-like domain; orange: predicted transmembrane domain; yellow: predicted protein kinase domain. The red rectangle shows the invariant catalytic motif RDxxxxN, whose conserved residues are indicated by red arrows.

***
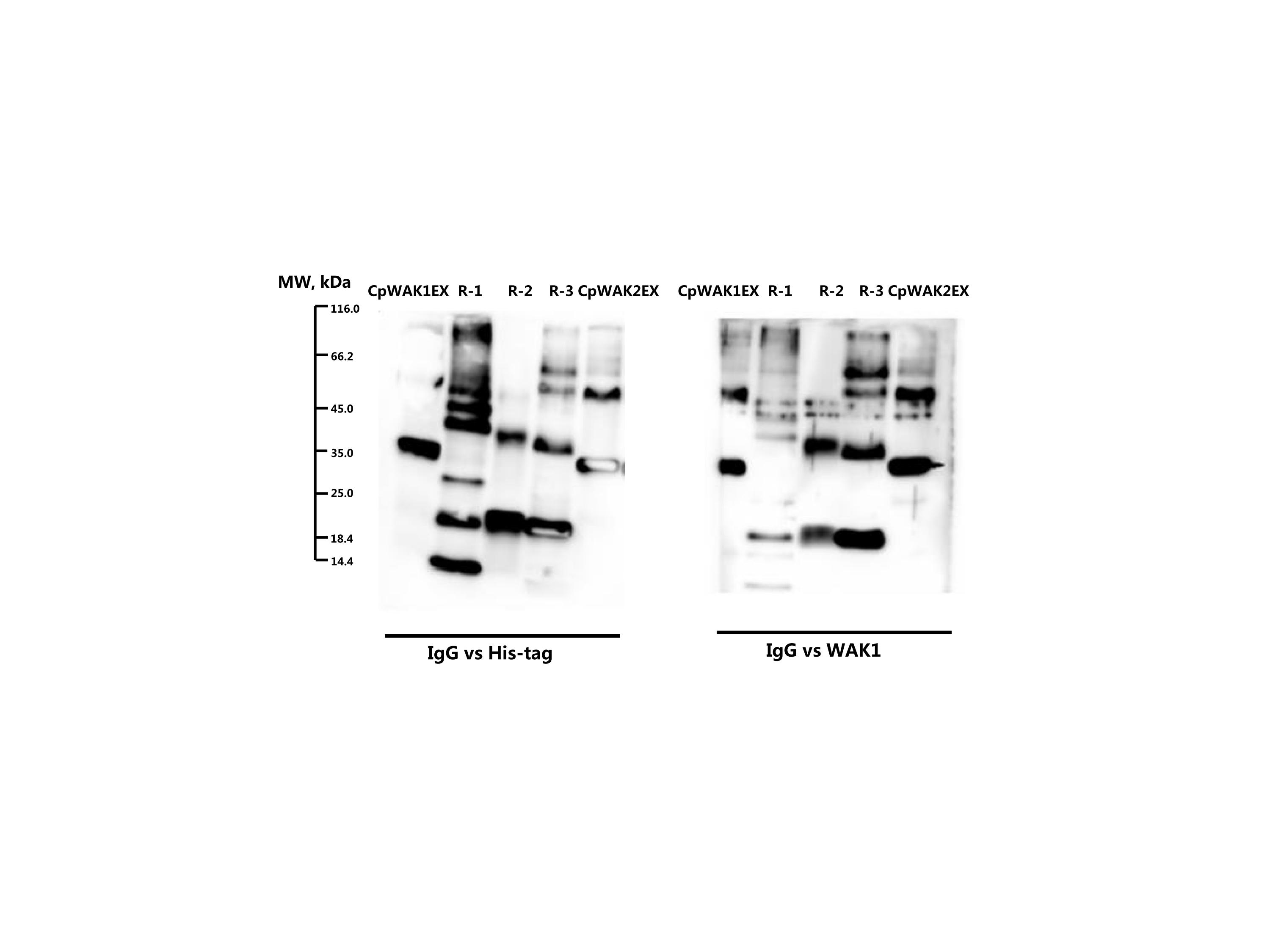
***

**Fig. S3** The formation of CpWAK multimers. All CpWAK fusion proteins were detected by the polyclonal anti-His-tag antibody (1:1000) (Invitrogen) and the polyclonal anti-CpWAK1 antibody (1:5000).


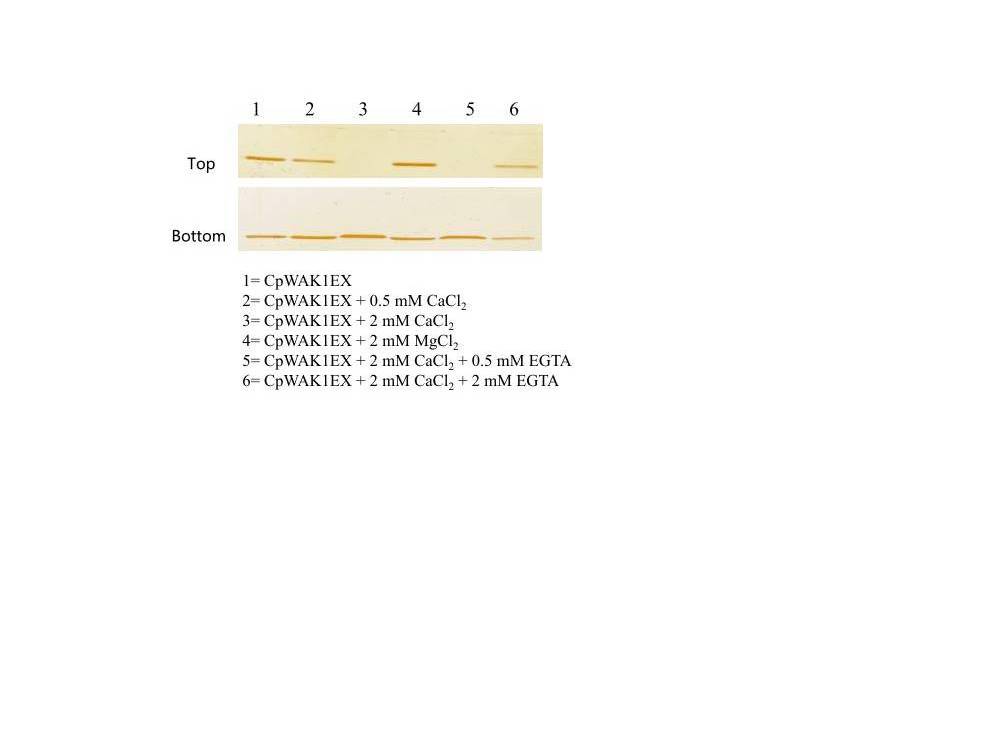


**Fig. S4** CpWAK1EX forms aggregates in the presence of Ca^2+^. The recombinant protein CpWAK1EX is precipitated with Ca^2+^ in Tris/NaCl buffer (pH 8.0). 50 ng of the purified recombinant protein CpWAK1EX were incubated in 30 μL of Tris/NaCl buffer (pH 8.0) with/without Ca^2+^, Mg^2+^ or EGTA for 1 h at RT. After centrifugation for 15 min (10,000 *g* RT), the top 10 μL and bottom 10 μL of the samples were pipetted out, separated by SDS-PAGE and then proteins were visualized by silver staining.

**
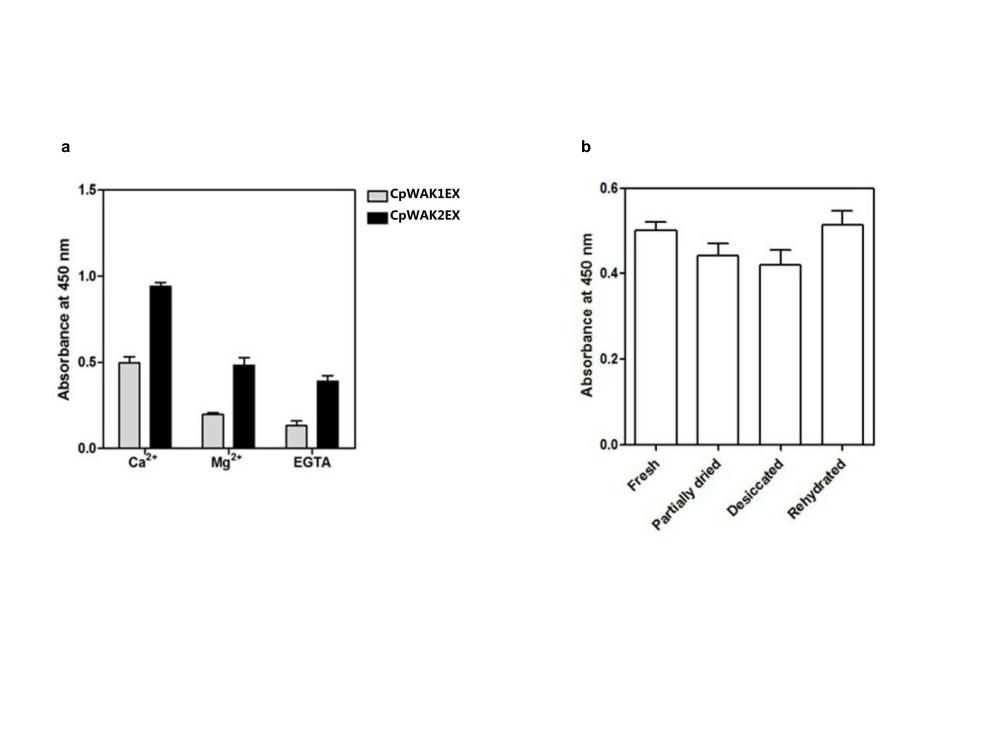
**

**Fig. S5 a**, Ca^2+^ facilitates the binding between pectin extracts with the CpWAKs extracellular domain in Tris/NaCl buffer (pH 8.0). **b**, CpWAK1 showed similar binding capacity to the pectin extracts prepared from fresh, partially dried, desiccated, and rehydrated *C. plantagineum* leaves. Statistical analysis was performed using one-way ANOVA (*P* = 0.097, not significant).


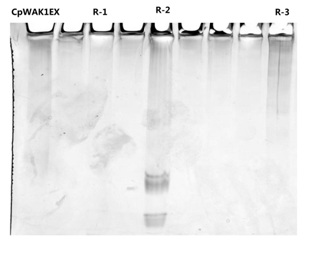


**Fig. S6** The migration of the truncated fragments of CpWAK1 in the SDS-PAGE gels without DTT.


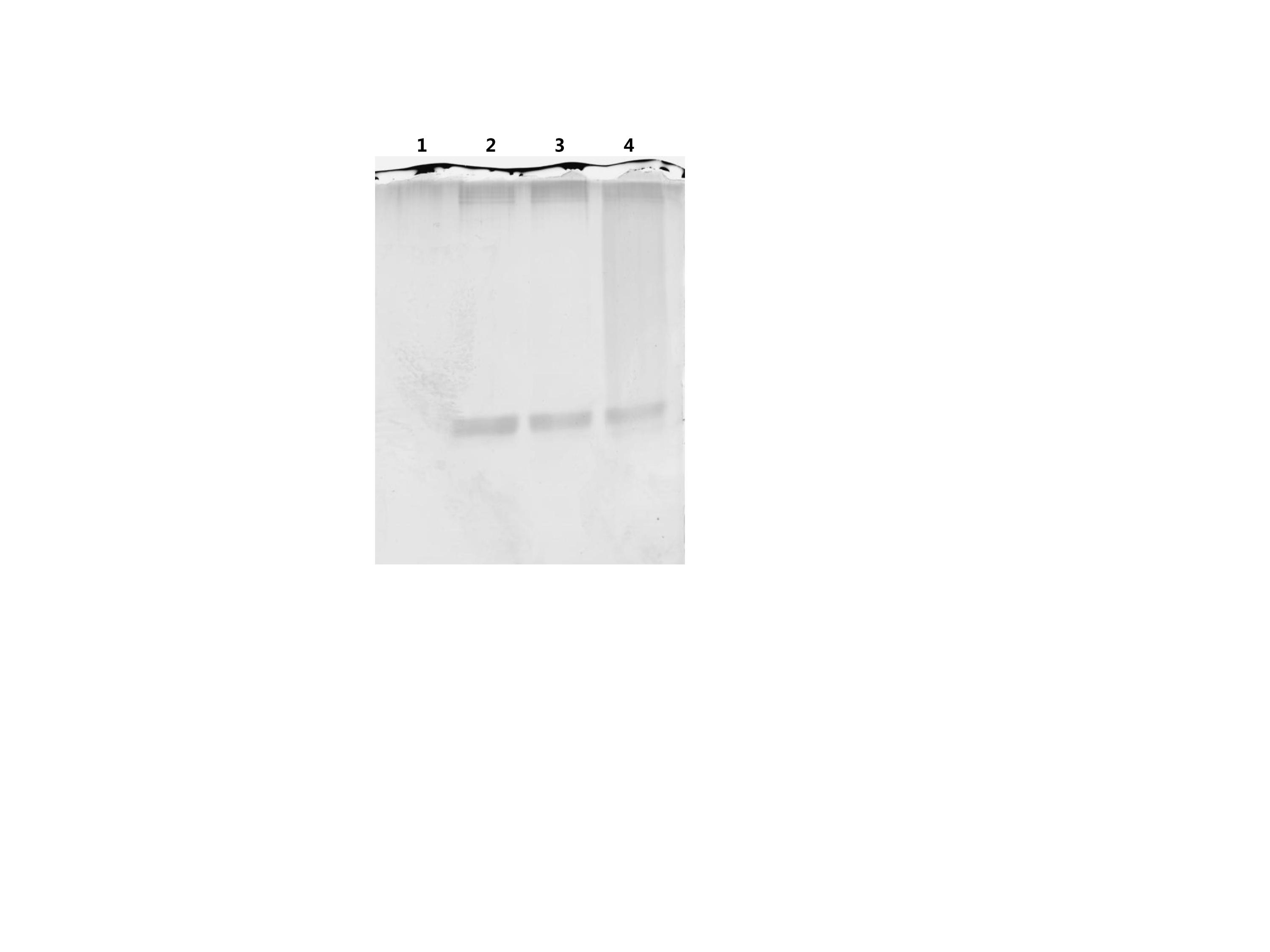


**Fig. S7** Protein band shift assays with CpWAK1EX and pectins. 0.2 µg of purified recombinant CpWAK1EX is incubated with/without 0.2 µg of citrus pectin in Tris/NaCl buffer (pH 8.0) with/without Ca^2+^ for 4 h at RT. Then the samples were separated by the SDS-PAGE gel with or without DTT. 1: CpWAKEX1 without DTT, 2: CpWAKEX1+DTT, 3: CpWAKEX1 incubated without pectin in Tris/NaCl buffer with Ca^2+^ + DTT, 4: CpWAKEX1 incubated with pectin in Tris/NaCl buffer with Ca^2+^ + DTT.
